# Enhanced Anti-Cancer Potential: Investigating the Combined Effects with *Coriolus versicolor* Extract and Phosphatidylinositol 3-Kinase Inhibitor (LY294002) In Vitro

**DOI:** 10.3390/ijms26041556

**Published:** 2025-02-12

**Authors:** Tomasz Jędrzejewski, Justyna Sobocińska, Bartosz Maciejewski, Marcela Slovakova, Sylwia Wrotek

**Affiliations:** 1 Department of Immunology, Faculty of Biological and Veterinary Sciences, Nicolaus Copernicus University, Lwowska 1 Str., 87-100 Toruń, Poland; j.sobocinska@umk.pl (J.S.); bartosz.maciejewski@umk.pl (B.M.); wrotek@umk.pl (S.W.); 2 Department of Biological and Biochemical Sciences, Faculty of Chemical Technology, University of Pardubice, Studentska 573 Str., 532 10 Pardubice, Czech Republic; marcela.slovakova@upce.cz

**Keywords:** *Coriolus versicolor*, phosphatidylinositol 3-kinase, LY294002, cancer

## Abstract

*Coriolus versicolor* (CV), known in traditional Chinese medicine for over 2000 years, is currently used in China and Japan to reduce chemotherapy or radiotherapy side effects in cancer patients. Despite extensive research, its effects still need improvement. This study aimed to determine if combining CV extract with LY294002, an inhibitor of the phosphatidylinositol-3-kinase (PI3K) signalling pathway, enhances cancer cell treatment, potentially leading to a novel therapeutic approach. Three human cancer cell lines (MCF-7, HeLa, and A549) were treated with CV extract alone or combined with LY294002. Cell viability was assessed using MTT assays. Then, HeLa and MCF-7 cells most sensitive to the co-treatment were used to evaluate colony formation, apoptosis, cell cycle, cell migration and invasion, and phospho-PI3K expression. The results demonstrated that LY294002 enhanced the CV extract’s anti-tumour effects by reducing cell viability and colony formation. The combined treatment with CV extract and LY294002 more effectively induced G0/G1 cell cycle arrest, promoted apoptosis, reduced cell invasion and migration, and inhibited phospho-PI3K expression compared to each agent alone. This study highlights the potent cytotoxic enhancement between CV extract and LY294002 on cancer cells, primarily by inhibiting phospho-PI3K expression. These findings suggest promising avenues for developing novel combination therapies targeting cancer.

## 1. Introduction

*Coriolus versicolor* (L.) Quél. (1886) (CV), also known as *Trametes versicolor* (L.) Lloyd (1920) mushroom, is one of the most investigated species, and has been used in traditional Chinese herbal medicine for over 2000 years. Ancient Chinese CV formulation is believed to promote health, strength, and longevity. Within traditional Chinese medicine, CV mushroom is believed to aid in detoxification, bolstering strength, optimizing liver and spleen functionality, and augmenting the immune system’s response, particularly when it’s dried, ground, and brewed into tea [1].

Nowadays, it is well-established that CV mushroom possesses various beneficial properties, including anti-oxidant, hypoglycaemic, anti-inflammatory, anti-viral, immuno-stimulating, and liver protection properties. These indicate its potential application value in treating liver disease, diabetes, arteriosclerosis, Alzheimer’s disease, cardiovascular and cerebrovascular diseases, osteoarthritis, inflammatory bowel disease, and many others [2,3,4,5]. However, the most widely studied properties of the compounds derived from CV mushroom are their anti-cancer activity. Numerous in vitro and in vivo studies have shown that CV compounds induce anti-tumoricidal effects against multiple cancer cell lines, inhibit tumour growth and metastasis in animal models, and display specific anti-angiogenic properties, which have been widely discussed in several review papers [3,5,6,7]. Clinical trials have reported both the indirect anti-cancer properties of CV through immuno-stimulating mechanisms and its direct anti-cancer activity, leading to the adoption of CV compounds as an adjunct therapy for cancer treatment in Japan and China. The bioactive compounds derived from CV, recommended for patients undergoing or post-radiation and chemotherapy, enhance their chances of survival, mitigate the immunosuppressive impacts of standard treatments, and alleviate symptoms associated with cancer therapy, such as fatigue, decreased appetite, vomiting, and pain [8,9,10,11,12]. Although CV extract is helpful in cancer treatment, it is not potent enough to be used as monotherapy. Hence, it is necessary to identify a substance that can enhance its effectiveness.

The present study investigated the effect of the combined treatment of cancer cells with CV extract and LY294002, a chemical inhibitor of the phosphatidylinositol-3-kinase (PI3K) signalling pathway. It is well-established that the PI3K pathway is frequently over-activated in human cancers, playing a significant role in carcinogenesis, tumour cell proliferation, invasion, and metastasis [13]. Over recent decades, research has concentrated on crafting PI3K inhibitors, aiming at individual or multiple proteins, progressing from preclinical tool compounds to specific medications for cancer patients [14]. Among them, LY294002 is a morpholine-containing compound which causes the induction of apoptosis in tumour cells, inhibits the invasiveness of cancer cells, and has anti-angiogenic properties [15,16]. The treatment with a combination of LY294002 and other drugs, such as tamoxifen [17], talazoparib [18], sorafenib [19], or rapamycin [20], is still under investigation, since this combination could both overcome the toxicity associated with LY294002 and may sensitise cancer cells to these drugs.

This study aimed to determine if combining CV extract with LY294002 can enhance cancer cell treatment, potentially leading to a novel therapeutic approach. For the experiments, we selected cell lines representing the most prevalent cancers worldwide in 2022, based on the latest World Health Organization (WHO) data. A549 cells were chosen as a model for lung cancer, the most commonly diagnosed cancer, while MCF-7 cells represent female breast cancer, which ranks second in global incidence [21]. Moreover, we included HeLa cells, one of the most extensively studied and well-documented cancer cell lines, facilitating comparative analysis and reproducibility across different studies.

## 2. Results

### 2.1. LY294002 and CV Extract Have Additive Cytotoxic Effects Against Cancer Cells

**Figure 1:**
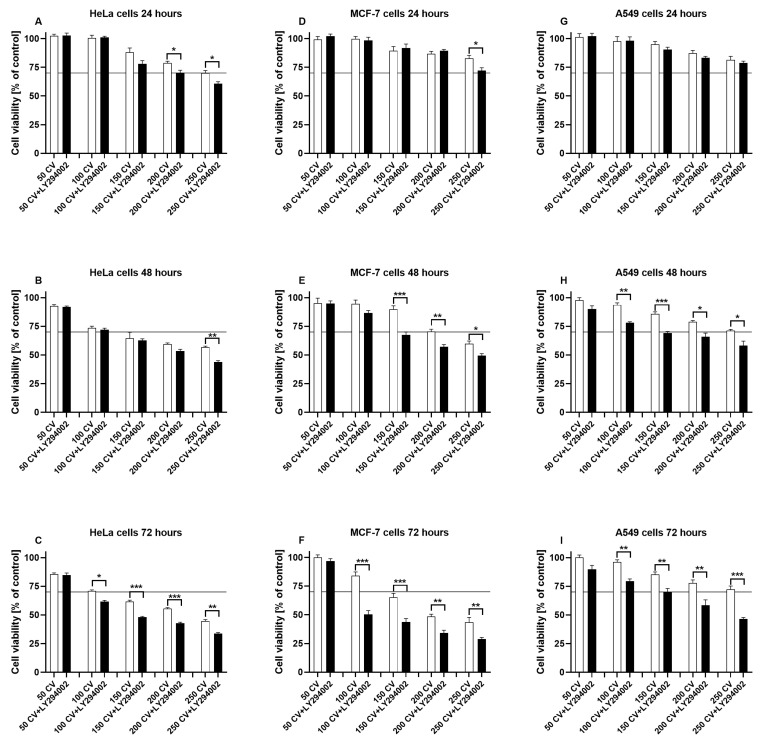
The viability of HeLa (**A**–**C**), MCF-7 (**D**–**F**), and A549 (**G**–**I**) cells stimulated with CV extract (50–250 µg/mL) or co-treated with the CV extract and LY294002 (10 µM) for 24, 48, and 72 h. The results are expressed as means ± S.E.M. of untreated cells from three independent experiments. Asterisks indicate significant differences between CV extract-treated and co-treated cells (* *p* < 0.05; ** *p* < 0.01; *** *p* < 0.001). Horizontal lines mark the 70% viability threshold for cytotoxicity.

The effect of LY294002 and CV extract on cancer cells was assessed through MTT and colony-forming assays. As shown in Figure 1, the viability of HeLa (Figure 1A–C), MCF-7 (Figure 1D–F), and A549 (Figure 1G–I) cells was decreased following the treatment with the CV extract in a dose- and time-dependent manner. The co-stimulation of cells with CV extract and LY294002 significantly reduced cell survival compared to the CV extract-treated cells. It was mainly observed after 72 h of stimulation when this additive effect was noticed for all tested doses of the CV extract and all used cell lines, except for a dose of 50 µg/mL. Since the CV extract at a 100 µg/mL concentration was the highest non-toxic dose according to the International Organization for Standardization (ISO) 10993-5 norm [22], it was used for further experiments. Moreover, LY294002 at a concentration of 10 µM was selected for the cell co-treatment, since this dose did not reduce cell viability below 70% (Figure S1 in the Supplementary Materials).

The half inhibitory concentrations (IC_50_ values) calculated based on the results from the MTT assay provided more detailed information about the differences in the cytotoxicity of the tested compounds (Table 1). These results show that the co-treatment of HeLa and MCF-7 cells with the CV extract and LY294002 (at a non-toxic dose) more effectively inhibited cancer cell survival than stimulation of cells only with the extract. This effect was observed for HeLa cells after 48 and 72 h of stimulation (*p* < 0.01 and *p* < 0.001, respectively) and for MCF-7 cell treatment after 72 h (*p* < 0.001). In contrast, the IC_50_ values calculated for A549 cells revealed that stimulation of these cells with CV extract in the presence of LY294002 did not significantly improve the cytotoxic effect compared with the cells treated only with CV extract. Therefore, the rest of the experiments were only conducted using HeLa and MCF-7 cells.

**Figure 2:**
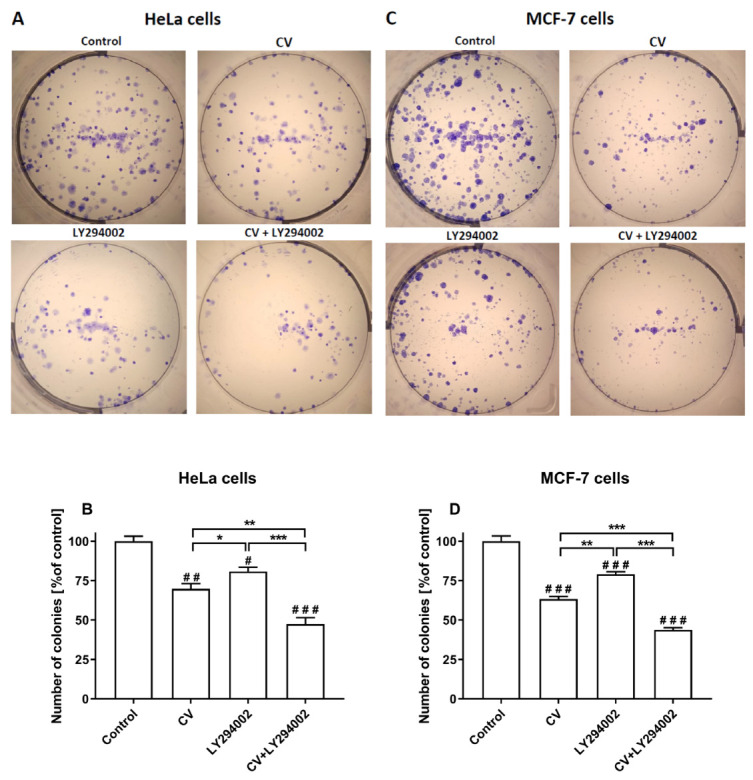
Colony numbers of HeLa (**A**,**B**) and MCF-7 (**C**,**D**) cells stimulated with CV extract (100 µg/mL), LY294002 (10 µM), or their combination. Colony formation was quantified as a percentage of untreated cells and presented as the means ± S.E.M. from three independent experiments. Asterisks show differences between the variants of cell treatment as indicated (* *p* < 0.05; ** *p* < 0.01; *** *p* < 0.001). Hashes show differences between control and treated cells (# *p* < 0.05; ## *p* < 0.01; ### *p* < 0.001).

The colony-forming assay was performed to evaluate the survival of cancer cells, measuring their capacity to form colonies from single cells. As shown in Figure 2, the stimulation of both HeLa and MCF-7 cells with the CV extract alone or LY294002 alone significantly inhibited colony formation compared to control cells. However, combining the CV extract and LY294002 provoked the lowest percentage of colony formation noticed for HeLa cells and MCF-7 cells.

### 2.2. Co-Treatment of Cancer Cells with LY294002 and CV Extract Increases Cell Cycle Arrest at the G0/G1 Phase

**Figure 3:**
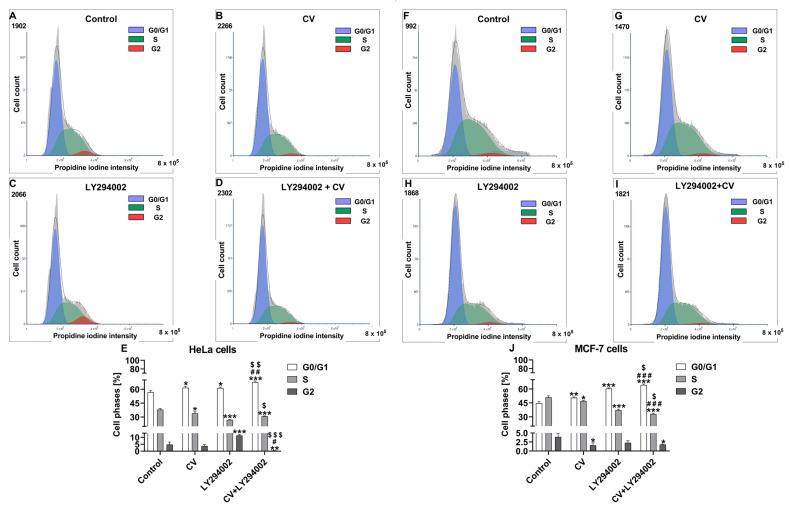
Cell cycle distribution of HeLa cells (**A**–**E**) and MCF-7 cells (**F**–**J**) treated with the CV extract (100 µg/mL), LY294002 (100 µM), or their combination compared with untreated cells (control). Representative histograms in (**A**–**D**) and (**F**–**I**) show the percentage of cells in G0/G1, S, and G2 phases. (**E**,**J**) present the cell cycle distribution as means ± S.E.M. from three independent experiments. Asterisks indicate differences between control and treated cells (* *p* < 0.05; ** *p* < 0.01; *** *p* < 0.001). Hashes show differences between CV extract and co-treated cells (# *p* < 0.05; ## *p* < 0.01; ### *p* < 0.001). $ marks present differences between LY294002 and co-treated cells (^$^ *p* < 0.05; ^$$^ *p* < 0.01; ^$$$^ *p* < 0.001).

Cell cycle analysis was performed to further clarify the mechanism of additive cytotoxic properties of CV extract and LY294002 inhibitor against cancer cells. The results showed that, in comparison with control cells, the single treatments led to a significant increase in the percentage of both MCF-7 and HeLa cells in the G0/G1 phase both after stimulation with CV extract (*p* < 0.01 and *p* < 0.05, respectively) as well as LY294002 (*p* < 0.001 and *p* < 0.05, respectively). However, the use of the combination of these agents significantly enhanced cell cycle arrest at the G0/G1 phase in comparison with either CV extract alone (*p* < 0.001 for MCF-7 cells and *p* < 0.01 for HeLa cells, respectively) or LY294002 alone (*p* < 0.05 for MCF-7 cells and *p* < 0.01 for HeLa cells, respectively). At the same time, the stimulation of both cell lines with CV extract alone or LY294002 alone resulted in a significant decrease in the S cell population phase compared to control cells (*p* < 0.05 and *p* < 0.001, respectively). However, this effect was the most noticeable in the co-treated cells. Finally, the decreased percentage of MCF-7 cells in the G2 phase was observed after stimulation with CV extract alone (*p* < 0.05) or a combination of CV and LY294002 (*p* < 0.05). In contrast, the G2 HeLa cell population was increased after CV extract treatment (*p* < 0.001) and decreased after cell co-stimulation (Figure 3).

### 2.3. Co-Treatment of Cancer Cells with LY294002 and CV Extract Increases Cell Apoptosis

**Figure 4:**
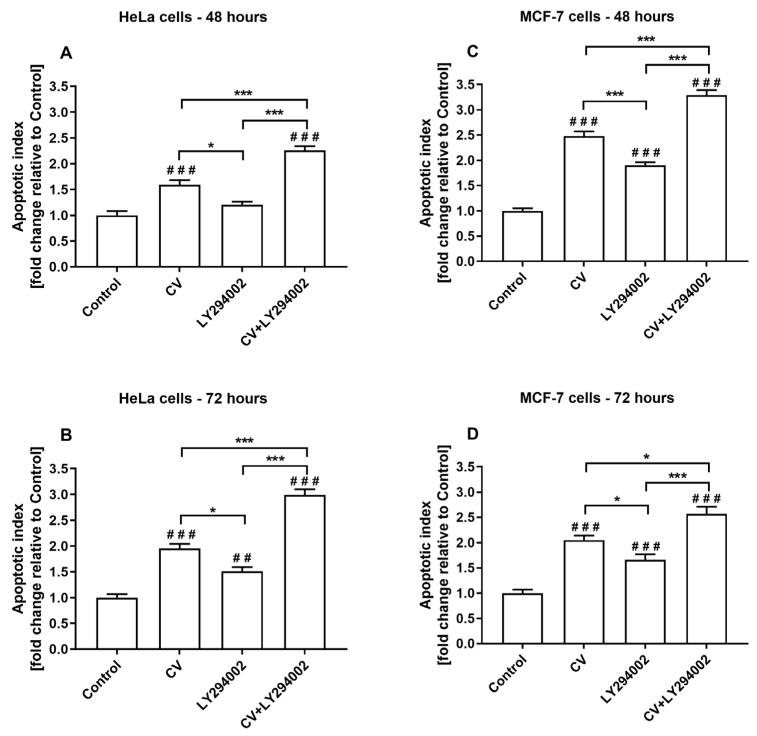
The induction of apoptosis in HeLa (**A**,**B**) and MCF-7 (**C**,**D**) treated with the CV extract (100 µg/mL), LY294002 (10 µM), or co-stimulated with both agents for 48 and 72 h. The level of the apoptotic index was expressed as a fold change relative to untreated cells (control; served as 1) and is presented as the means ± S.E.M. Asterisks indicate differences between the variants of cell stimulation as indicated (* *p* < 0.05; *** *p* < 0.001). Hashes present differences between control cells and the cells treated with the agents (## *p* < 0.01; ### *p* < 0.001).

The apoptosis analysis was performed using the cell death detection ELISAPLUS assay, which allows for quantifying histone-complexed DNA fragments out of the cytoplasm of the apoptotic cells. This analysis elucidated the underlying mechanism of the additive inhibition of the growth of HeLa and MCF-7 cancer cells co-stimulated with CV extract and LY294002. The results showed that the treatments of both cell lines only with the CV extract significantly increased the level of apoptosis, which was observed after 48 and 72 h (Figure 4). A similar effect was noticed for the cells stimulated with LY294002 alone, except for the HeLa cells treated with this inhibitor for 48 h. Moreover, the increase in nucleosome release in LY294002-treated cells was lower than the values calculated for the cells stimulated with the CV extract only. However, combining the CV extract and LY294002 induced the highest level of apoptosis, which was observed in HeLa cells after 72 h and MCF-7 cells after 48 h.

### 2.4. LY294002 Increases the Anti-Migratory Activity of the CV Extract

**Figure 5:**
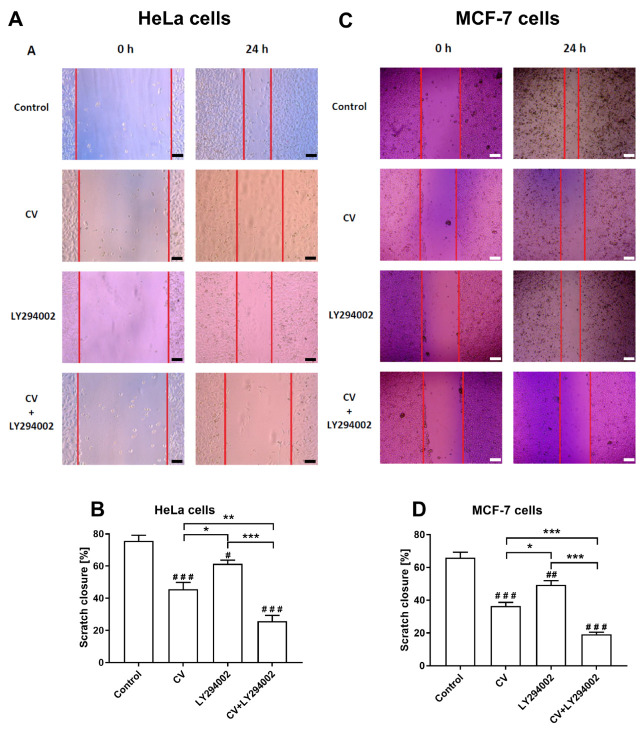
Anti-migratory effect of CV extract (100 µg/mL), LY294002 (10 µM), or their combination on HeLa (**A**,**B**) and MCF-7 (**C**,**D**) cells compared to control cells. (**A**,**C**) show the representative cell images at 0 h and after 24 h, respectively. Black and white lines in the lower corners represent a scale of 200 µm. (**B**,**D**) present the quantitative scratch closure (%) from 0 h to 24 h. Data are presented as mean ± S.E.M. from three independent experiments. Asterisks indicate significant differences between treatments (* *p* < 0.05; ** *p* < 0.01; *** *p* < 0.001). Hashes show differences between control and treated cells (# *p* < 0.05; ## *p* < 0.01; ### *p* < 0.001).

To investigate the anti-migratory activity of the tested agents, scratch assays were performed to estimate the cell migration levels. As shown in Figure 5, although the use of CV extract alone or LY294002 alone decreased cell migration, the combined cell treatment with these two agents most effectively inhibited the migration of HeLa cells and MCF-7 cells. Moreover, both HeLa and MCF-7 cells stimulated only with LY294002 demonstrated higher levels of scratch closure than the CV extract-treated cells.

### 2.5. LY294002 Decreases the Invasive Ability of the Cancer Cells Stimulated with the CV Extract

**Figure 6:**
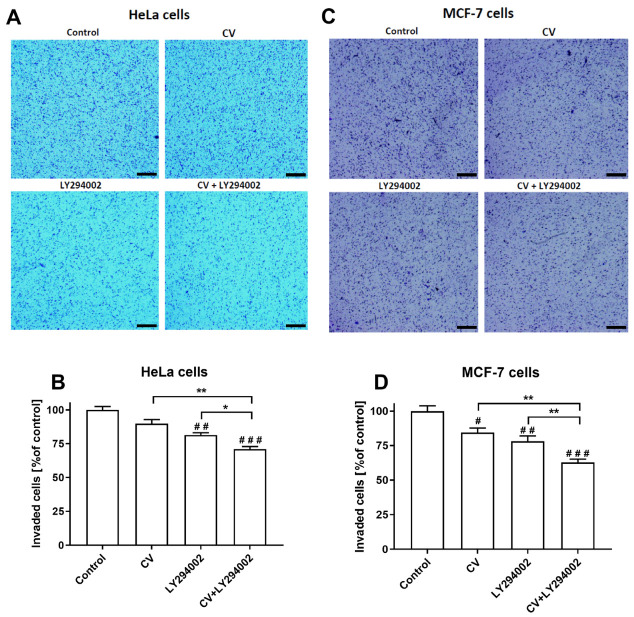
Invasive ability of HeLa cells (**A**,**B**) and MCF-7 cells (**C**,**D**) stimulated with CV extract (100 µg/mL), LY294002 (10 µM), or their combination compared to control cells. (**A**,**C**) show representative images of invaded cells, while (**B**,**D**) display quantitative evaluations (% of control). Black lines in the lower corners represent a scale of 200 µm. Data are presented as mean ± S.E.M. from three independent experiments. Asterisks indicate significant differences between treatments (* *p* < 0.05; ** *p* < 0.01). Hashes show differences between control and treated cells (# *p* < 0.05; ## *p* < 0.01; ### *p* < 0.001).

The results of the transwell tumour cell invasion assays showed that the CV extract at a concentration of 100 µg/mL significantly decreased the invasive ability of only MCF-7 cells (*p* < 0.05) compared to control cells, without an effect on HeLa cells. In contrast, the number of HeLa and MCF-7 cells invaded after the stimulation only with LY294002 was lower than control cells (*p* < 0.01). However, the combined treatment with the CV extract and LY294002 most significantly reduced the invasive ability of HeLa cells and MCF-7 cells (*p* < 0.001) (Figure 6).

### 2.6. Co-Treatment of Cancer Cells with LY294002 and CV Extract Potentiated Inhibition of Phospho-PI3K Expression

**Figure 7:**
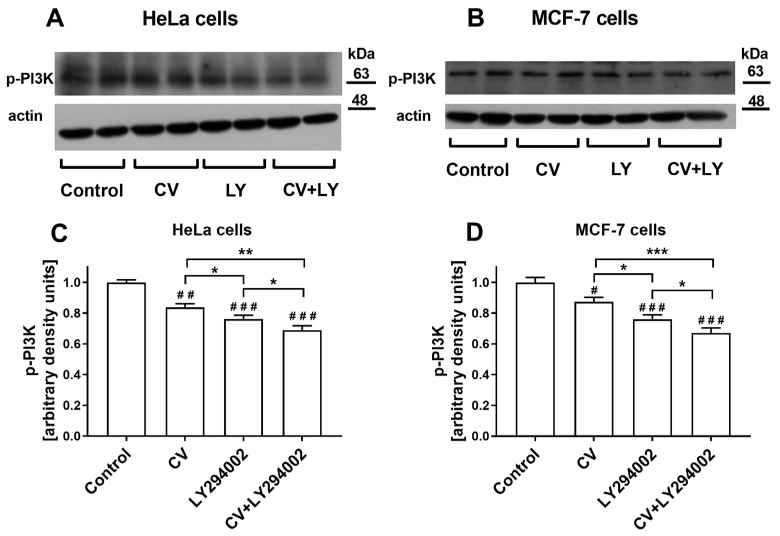
Representative Western blot images of p-PI3K and actin expression in untreated (control) HeLa (**A**) and MCF-7 cells (**C**) or cells stimulated with CV extract (100 µg/mL), LY294002 (10 µM), or both for 24 h. Densitometric bar graphs show p-PI3K levels in cell lysates (**B**,**D**). Data are presented as mean ± S.E.M. Asterisks indicate significant differences between treatments (* *p* < 0.05; ** *p* < 0.01; *** *p* < 0.001). Hashes show differences between control and treated cells (# *p* < 0.05; ## *p* < 0.01; ### *p* < 0.001).

The Western blot analysis was applied to check whether the bilateral effect of cancer cell co-stimulation with the CV extract and LY294002 results from changes in the phospho-PI3K (p-PI3K) expression. The results showed that the CV extract decreased the expression of p-PI3K in both cancer cell lines compared to control cells (*p* < 0.01 for HeLa cells and *p* < 0.05 for MCF-7 cells). However, this effect was weaker than in the cells stimulated only with LY294002 (*p* < 0.05 for both cancer cell lines). The lowest level of p-PI3K expression was observed in the cancer cells co-treated with both compounds for 24 h (Figure 7).

## 3. Discussion

Out of over 270 species of mushrooms known for their immunomodulatory properties, 50 are identified as non-toxic and have undergone testing in animal models. However, human cancer studies have investigated only six of these species. Notably, the *Coriolus versicolor* mushroom stands out as the sole species studied in phase I, II, and III clinical trials involving patients with stomach, colorectal, oesophageal, and breast cancers [23]. The promising application of CV mushroom in cancer therapy stems from its capacity to discriminate between cancerous and normal cells, induce different modes of cancer cell death, such as apoptosis, necrosis, or necroptosis, and enhance the host immune response to cancer, which has been widely discussed in several review papers [3,5,6,7]. Moreover, numerous in vitro and in vivo studies show that active compounds derived from CV can induce anti-tumour effects when used as a monotherapy, and the cancer cell sensitization to various chemotherapeutic agents by CV has also been widely studied.

Despite promising results, there is still a need to refine therapy using CV by introducing an additional component because its anti-cancer effectiveness was observed only at high doses, which is manifested by high IC_50_ values observed for certain cancer cell lines, such as melanoma, breast cancer, leukaemia, stomach cancer, and colorectal cancer [5,24,25,26,27]. On the other hand, the use of CV in combination with chemotherapy has disadvantages related to side effects induced by chemotherapeutics that, unlike CV, do not differentiate cancerous cells and normal cells. Therefore, solutions capable of potentiating the anti-tumour value of CV mushroom in combined treatment in oncology patients, are still being searched for.

This study aimed to explore the therapeutic efficacy of co-treating cancer cells with the PI3K inhibitor (LY294002) and CV extract. The PI3K signalling pathway encompasses a range of biological processes pertinent to malignant cancers, such as cell proliferation, differentiation, invasion, and metastasis. The aberrant activation of this pathway also holds significant sway over the emergence of multidrug resistance in various types of neoplasms, representing a primary hurdle in chemotherapy efficacy [28]. Although several PI3K signalling inhibitors have been influential in the inhibition of tumour progressions during clinical trials and approved by the Food and Drug Administration in the USA, most of them have demonstrated only modest clinical efficiency as monotherapies because of drawbacks in their pharmacokinetics and tolerability [29,30]. We have chosen to explore the impact of a PI3K inhibitor on CV-induced effects for two main reasons. Our previous findings suggested that CV extract mediates immunostimulatory properties related to cytokine production in macrophages through the PI3K signalling pathway [31]. Secondly, there is a lack of research examining the potential impact of CV extract on cancer cells via this pathway.

Previous results from MTT assays showed that CV extract decreased cancer cell viability, confirming the results widely discussed in many papers [3,5,7,32]. In the present paper, we have also demonstrated that this extract reduced cancer cell colony formation, indicating that CV extract may suppress the regrowth and recurrence of tumours after treatment [33]. Significantly, the use of LY294002 inhibitor at a non-cytotoxic dose improved the anti-tumour properties of the CV extract compared to the anti-cancer activity of the CV extract alone, which was manifested by the decreased cell survival rate (IC_50_ values) and reduced ability of single cancer cells to survive and reproduce to form colonies.

Additionally, we investigated cell cycle distributions to further explain the underlying mechanism of the additive toxic effect resulting from treating cancer cells with the CV extract and LY294002 combination. The results showed that, compared to control cells, the treatments with CV extract alone or LY294002 alone led to a significant increase in the percentage of cancer cells in the G0/G1 while causing a significant decrease in the S cell population phase. These discoveries align with prior research, which has shown that CV extract disrupts the progression of the cancer cell cycle and induces cell cycle arrest at the G0 [34], G0/G1 [35], G1/S [36], and G2/M [37] phases. Likewise, LY294002 is regarded as a promising therapeutic agent due to its ability to inhibit the growth of different cancer cell types, primarily by inducing cell cycle arrest at both the G0/G1 [38] and G1/M [17] phases. However, our results demonstrated that combined treatment of cancer cells with CV extract and LY294002 notably intensified cell cycle arrest at the G0/G1 phase in cancer cells compared to using each compound alone.

The G0/G1-phase arrest of cell cycle progression is widely recognised as a crucial juncture where cells can either engage in repair mechanisms or proceed along the apoptotic pathway. Apoptosis, a significant form of cell death triggered by cytotoxic effects, is thus facilitated by this arrest [39]. Numerous experiments also show that CV extract [35,40,41,42] and LY294002 [17,43] can induce the apoptosis of cancer cells. Based on these findings and the results from the cell cycle distributions, we decided to check how combined treatment with CV extract and LY294002 affects the apoptotic death of cancer cells. Our results showed that co-treatment with the CV extract and LY294002 more effectively induced the apoptosis of cancer cells than the stimulation with each of these agents alone. This effect can be partially associated with the more effective arrest of the cancer cell cycle at the G0/G1 phase observed during the combined treatment of cancer cells [17].

Cancer cells’ migration and invasion capabilities facilitate their movement and infiltration into lymphatic and blood vessels, enabling the spread of neoplastic cells and the development of metastatic growth in distant organs. Therefore, controlling cancer cell migration and invasion becomes a pivotal focus of cancer treatment [44]. The aggressive nature of cancer cells is governed by an intricate network of signalling pathways that oversee crucial functions, including growth, survival, migration, and invasion. The PI3K signalling pathway has been causally linked to these four responses. Moreover, overexpression of PI3K in a wide range of human tumours is detected and often linked with poor prognosis [13]. Accumulating evidence suggests that a PI3K inhibitor named LY294002 can significantly attenuate the invasion and migration of leukaemia cells [45], prostate cancer cells [46], oral squamous carcinoma cells [47], osteosarcoma cells [48], and pancreatic cancer cells [49]. Here, we illustrated that LY294002 also exhibits these properties when tested against breast and cervical cancer cells. There are also some experimental data showing that CV extract has anti-migratory and anti-invasive potentials against numerous tumour cells, including triple-negative breast cancer [42], oestrogen receptor (ER)-positive breast cancer [26], colon cancer [25], pancreatic and gastric cancer [50], and melanoma [51]. Our results demonstrated that CV extract also inhibits the invasiveness and migration of cervical cancer cells. The combined treatment with the CV extract and LY294002 significantly reduced the invasive and migratory activities of ER-positive breast and cervical cancer cells more considerably than the cell stimulation with each compound alone. Additional investigations are imperative to clearly elucidate the mechanism behind this enhanced interaction. However, we suppose that it can result from the effect of both compounds on the simultaneous inhibition of the PI3K pathway. Our assumption is confirmed by the results from the Western blot analysis, which showed that co-treatment of cells with the CV extract and LY294002 more significantly inhibited the protein expression of p-PI3K in the cancer cells than LY294002 alone. We also observed that CV extract decreased p-PI3K expression in cancer cells for the first time compared to control cells.

Additional studies are required to assess the effects of combined stimulation with CV extract and LY294002 on normal cells, providing a deeper understanding of their impact on healthy tissues. Numerous studies, including ours, have shown that CV extract not only lacks toxic effects on normal cells but that it has also been found to enhance the viability and proliferation of these cells. This mitogenic effect has been particularly observed in various immune cell types, including B and T lymphocytes, splenocytes, monocytes, macrophages, and dendritic cells [3,5]. Moreover, CV extract can increase the resistance of normal cells to chemotherapy-induced cytotoxicity, mainly by enhancing antioxidant activity and immune response. Studies investigating the co-treatment of CV extract and chemotherapeutics have revealed several beneficial effects, including protective effects on bone marrow cells and hepatocytes, as well as the enhancement of immune cell functions. These findings suggest that CV extract may help mitigate the side effects of chemotherapy while supporting the body’s natural defence mechanisms [5,10]. In contrast, treatment of normal cells with LY294002 can exhibit cytotoxic effects, particularly at high concentrations. This is primarily due to the inhibition of the PI3K pathway, which plays a crucial role in the survival, proliferation, and metabolic regulation of various normal cells [52]. Therefore, it is crucial to use relatively low doses of LY294002 to minimise toxicity in healthy cells while simultaneously enhancing the sensitivity of cancer cells to the cytotoxic effects of natural compounds, like CV extract.

## 4. Materials and Methods

### 4.1. Cell Culture

The MCF-7 breast cancer cells and lung cancer cell line A549 were procured from the European Collection of Authenticated Cell Cultures (ECACC; Salisbury, UK), while cervical cancer cells (HeLa) were sourced from the American Type Culture Collection (ATCC; Manassas, VA, USA). Each cell line was cultured in DMEM supplemented with 10% foetal bovine serum (FBS) at 37 °C in an atmosphere with 5% CO_2_. The reagents used for cell cultures were purchased from Merck KGaA (Darmstadt, Germany).

### 4.2. Preparation of CV Extract and LY294002 Solution

The CV extract was provided by the MycoMedica Company (Police nad Metují, Czech Republic). Previously, we demonstrated that CV extract contains polysaccharide peptide (PSP) and polysaccharide krestin (PSK), which are considered the most potent compounds for anti-tumour and immunomodulatory effects. Additionally, we have identified various low-molecular-weight compounds in the extract, including vitamins (D3, K, retinyl acetate), monosaccharides (arabinose, glucuronic acid), amino acids (histidine), fatty acids (palmitic acid, oleic acid, linolenic acid), and phenolic compounds (p-hydroxy benzoic acid) [53].

The culture medium was utilised to dissolve the CV extract, forming a 4 mg/mL stock solution, which was received by continuous agitation for 48 h at room temperature. The soluble supernatant, which held 1 mg of polysaccharide peptides, underwent sterilization via a 0.22 µm filter. Subsequently, it was diluted with culture medium to the specified concentrations as indicated.

The LY294002, an inhibitor of PI3K, was purchased from Cell Signalling Technology (Leiden, The Netherlands) and reconstituted in dimethyl sulfoxide (DMSO; Merck KGaA, Darmstadt, Germany). Before cell stimulation, the stock solution of the inhibitor was diluted to the desired concentration using a culture medium. Throughout the cell treatment, the ultimate concentration of DMSO remained below 0.1%.

### 4.3. Cell Viability

The cancer cells were seeded in a density of 3 × 10^3^ cells/well and pre-incubated overnight. Then, the cells were treated with the CV extract (50, 100, 150, 200, and 250 µg/mL) alone or co-stimulated with the CV extract and LY294002 (10 µM) for 24, 48, and 72 h. For the cell co-treatment, a 10 µM concentration of inhibitor was selected as it maintained cell viability above 70% (Supplemental Figure S1) and, therefore, it cannot be considered a cytotoxic agent according to the ISO 10993-5 norm [22]. After treatment, the MTT solution (0.5 mg/mL; Merck KGaA, Darmstadt, Germany) was added for 3 h, followed by dissolving formazan crystals with DMSO and measuring the optical density at 570 nm using a microplate spectrophotometer (Synergy HT; BioTek Instruments, Winooski, VT, USA). The results were presented as a percentage of the control cells. Using data from the MTT assays, the IC_50_ values were determined using GraphPad Prism 7.0 (GraphPad Software Inc., San Diego, CA, USA). For subsequent experiments, HeLa and MCF-7 cells were used, since their viability was decreased during co-stimulation with CV extract and LY294002 compared with the treatment with the extract alone. Furthermore, a 100 µg/mL CV extract concentration was selected, as it maintained viability above 70%.

### 4.4. Colony Formation Assay

The cells were seeded in 6-well plates (3 × 10^5^ cells/well) overnight, followed by the stimulation with the CV extract (100 µg/mL), LY294002 (10 µM), or their combination for 48 h. After trypsinization, the live HeLa and MCF-7 cells were seeded in 6-well plates (in triplicates) at a density of 2 × 10^2^ or 1 × 10^3^ cells/well. They were cultivated in 2 mL media for 10 or 14 days, respectively. Every three days, the media were exchanged with fresh culture medium. After the designated culture period, the cells were washed with PBS and fixed with 100% *v*/*v* methanol for 20 min at room temperature. Next, staining was carried out using a 0.5% *v*/*v* crystal violet solution (prepared in 25% *v*/*v* methanol) for 25 min; the colonies were then washed with water and we air-dried the plates before performing the colony counting. Colonies containing more than 50 cells were counted in triplicate.

### 4.5. Apoptosis Assay

The cell death detection ELISAPLUS assay (Roche Diagnostics, Mannheim, Germany) was used to quantify histone-complexed DNA fragments (mono- and oligonucleosomes) out of the apoptotic cells’ cytoplasm after the induction of apoptosis. HeLa and MCF-7 (1 × 10^4^ cells/well) were pre-incubated for 24 h, followed by treatment with the CV extract (100 µg/mL), LY294002 (10 µM), or co-stimulated with both agents for 48 and 72 h. The subsequent procedures of the sandwich enzyme-linked immunoassay followed the instructions provided by the manufacturer. The colour development, indicative of the number of nucleosomes captured in the antibody sandwich, was assessed at 405 nm (with a reference wavelength of 490 nm) using a Synergy HT Microplate Reader (BioTek Instruments, Winooski, VT, USA). The results were expressed as a fold change relative to untreated cells, normalised to 1.

### 4.6. Cell Cycle Analysis

The cell cycle was assessed following the manufacturer’s guidelines using the CellCycleFlowEx Kit (EXBIO Praha, a.s., Vestec, Czech Republic), which facilitates the quantification of DNA content through propidium iodide staining, followed by analysis using flow cytometry. The cells were seeded at a density of 3 × 10^5^ cells in a 25 cm^2^ tissue culture flask (in triplicates), cultured overnight, followed by starving with serum-free medium for 6 h. Then, the cells were treated with the CV extract (100 µg/mL), LY294002 (10 µM), or their combination for 48 h. For flow cytometry analysis, the cells were stained for 30 min with propidium iodide, and RNA was digested using RNAse A (EXBIO Praha, a.s., Vestec, Czech Republic). Finally, the cell cycle distributions were analysed using a BriCyte E6 flow cytometer (Mindray, Shenzhen, China) and FCS Express 7 Image Cytometry Software (DeNovo Software, Pasadena, CA, USA).

### 4.7. Scratch Assay

The cells (2 × 10^5^/well) were seeded in 12-well plates and pre-incubated in DMEM with 10% FBS until they reached 100% confluency. The cell monolayer was scratched (in triplicate) using a 10 µL pipette tip, and cells were treated with CV extract, LY294002, or both agents in DMEM with 1% FBS for 24 h. Cell migration into the scratched area was monitored using a Leica DMi1 inverted microscope with a digital camera (Leica Microsystems, Wetzlar, Germany), both at the initial moment and 24 h later. Migration scratch closure was measured with ImageJ software v1.54d (National Institutes of Health, Bethesda, MD, USA) and expressed as a percentage of the initial distance.

### 4.8. Invasion Assay

Cell invasion was assessed using the CHEMICON Cell Invasion Assay Kit (Merck KGaA, Darmstadt, Germany). Cells (3 × 10^5^/well) were seeded in 6-well plates overnight (in triplicates) and treated with CV extract, LY294002, or both agents for 48 h. Then, 5 × 10^4^ live cells were suspended in 300 µL serum-free DMEM and added to the upper chambers, while 500 µL DMEM with 20% FBS was added to the lower chambers. After 24 h, non-invasive cells were removed using a cotton swab, and invasive cells were stained with a staining solution provided by the manufacturer and imaged (40× magnification). The level of cell invasion was also quantitated by dissolving stained cells in 10% acetic acid (200 µL/well) in addition to taking colorimetric absorbance readings at 560 nm (Synergy HT Microplate Reader, BioTek Instruments, Winooski, VT, USA). The invasion levels were expressed as a percentage of untreated cells.

### 4.9. Western Blot Analysis

Western blotting was used to determine p-PI3K protein levels of in HeLa and MCF-7 cells after 24 h treatment with CV extract, LY294002, or both agents. After stimulation, the cells were washed with ice-cold PBS and lysed in RIPA buffer supplemented with a protease and phosphatase inhibitor cocktail (all reagents sourced from Merck KGaA, Darmstadt, Germany). The protein concentration within the lysates was assessed using a Pierce™ BCA Protein Assay Kit (Thermo Fisher Scientific, Waltham, MA, USA). The lysates were mixed with sample buffer and subjected to electrophoresis. Then, proteins were transferred to nitrocellulose membranes, and incubated with rabbit anti-phospho-PI3K p85 (cat. no. 4292; Lot: 1; Cell Signalling Technology, Leiden, The Netherlands) and mouse anti-actin (cat no. CP01; Lot: D00164515; Merck KGaA, Darmstadt, Germany) antibodies. Subsequently, the membranes were exposed to either goat anti-rabbit (cat. no. 612657; Lot: 0296526 MP Biomedicals; Santa Ana, CA, USA) or goat anti-mouse (cat no. 115-035-003; Lot no.: 152341; Jackson ImmunoResearch, Cambridge, United Kingdom) antibodies conjugated with horseradish peroxidase. Protein bands were visualised with SuperSignalWest Pico substrate (Thermo Fisher Scientific, Waltham, MA, USA) and analysed densitometrically using ImageJ software v1.54d.

### 4.10. Statistical Analyses

Statistical analyses were performed using GraphPad Prism 7.0 software (GraphPad Software Inc., San Diego, CA, USA). Data are presented as mean ± standard error of the mean (S.E.M.) and were assessed using one-way ANOVA followed by Tukey’s multiple comparisons test, with statistical significance set at *p* < 0.05.

## 5. Conclusions

In conclusion, while compounds derived from CV extract are utilised in complementary cancer treatment, there remains a quest for novel therapies to enhance its efficacy. Our study illustrates that combining CV extract with a chemical inhibitor of the PI3K signalling pathway induces an additive cytotoxic effect on cancer cells. This suggests a promising avenue for developing more effective anti-cancer therapies incorporating CV extract within conventional treatment protocols in China, Japan, and in Western countries. However, this study has certain limitations, including the need to compare the effects of CV extract and LY294002 not only on cancer cells but also on healthy cells to assess the potential cytotoxicity of this kind of cell treatment. Additionally, further research is required to examine the interactions between the two compounds and determine their nature, such as their synergistic or additive effects, using isobolographic analysis. Finally, future studies should incorporate animal models to evaluate the pharmacokinetics, bioavailability, and systemic effects of the CV extract and LY294002 combination.

## Figures and Tables

**Table 1:** The time-dependent inhibitory concentration of the CV extract and LY29402 (10 µM) against different cell lines in the MTT assay.

Time	Sample	IC_50_		
		HeLa	MCF-7	A549
24 h	CV	>250	>250	>250
	CV + LY294002	>250	>250	>250
48 h	CV	>250	>250	>250
	CV + LY294002	215.5 ± 7.6 **	239.4 ± 9.9	>250
72 h	CV	225.2 ± 11.2	208.6 ± 7.8	>250
	CV + LY294002	153.0 ± 4.5 ***	133.4 ± 3.9 ***	244.7 ± 6.7

Data are presented as mean ± S.E.M. IC_50_: half-maximal inhibitory concentration (µg/mL). Asterisks show significant differences between the co-stimulated cells and those treated only with CV extract (*** *p* < 0.001; ** *p* < 0.01).

## Data Availability

The original contributions presented in this study are included in the article/Supplementary Material. Further inquiries can be directed to the corresponding author.

## Supplementary Materials

The following supporting information can be downloaded at: https://www.mdpi.com/article/10.3390/ijms26041556/s1.

## Abbreviations

The following abbreviations are used in this manuscript:

CV	Coriolus versicolor
LY294002	phosphatidylinositol-3-kinase inhibitor
PI3K	phosphatidylinositol-3-kinase
p-PI3K	phospho- phosphatidylinositol-3-kinase
