# Supplementary material for: Enhanced Anti-Cancer Potential: Investigating the Combined Effects with Coriolus versicolor Extract and Phosphatidylinositol 3-Kinase Inhibitor (LY294002) In Vitro
Source: Int J Mol Sci. 2025 Feb 12;26(4):1556. doi: 10.3390/ijms26041556 (PMC11855823; doi:10.3390/ijms26041556)
Supplement: Supplementary file 1 [file ijms-26-01556-s001.zip › ijms-3476407-supplementary.pdf]

Synergistic Anti-Cancer Potential: Exploring Dual Drug Strategy with *Coriolus Versicolor* Extract and Phosphatidylinositol 3-Kinase Inhibitor (LY294002) *In Vitro*

Tomasz Jędrzejewski, Justyna Sobocińska, Bartosz Maciejewski, Marcela Slováková, Sylwia Wrotek

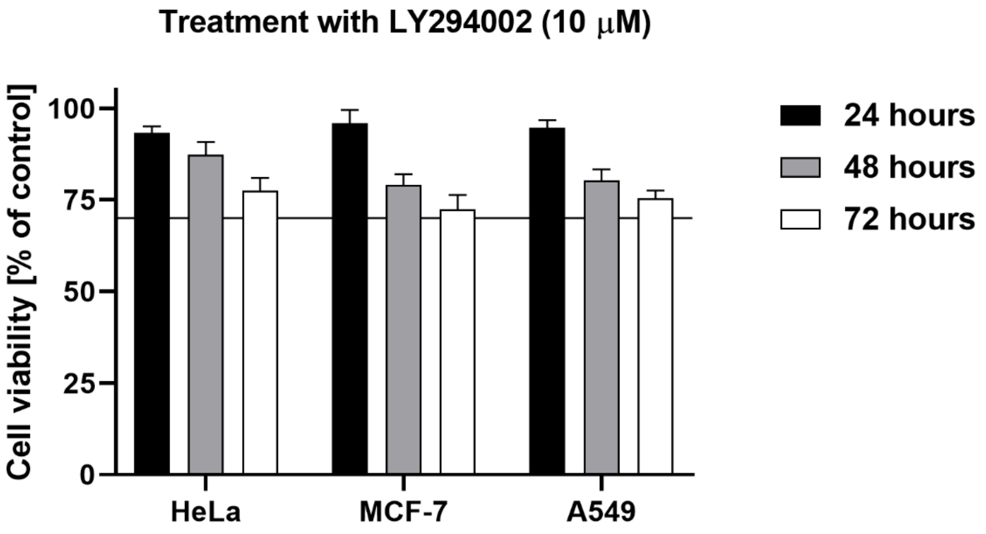

Figure S1. The viability of HeLa, MCF-7 and A549 cancer cells stimulated with LY294002 at a concentration of 10 µM for 24, 48 and 72 h. A horizontal line shows a potential cytotoxicity of the inhibitor, when the cell viability decreases below 70% according to ISO 10993-5 norm.
